# Physicochemical properties of ultrasound-pretreated pea starch and its inclusion complexes with lauric acid

**DOI:** 10.1016/j.fochx.2023.100879

**Published:** 2023-09-15

**Authors:** Liuyang Xiao, Yingtao Yu, Xiaofan Yang, Zhaojun Wei, Lihong Han

**Affiliations:** a Collaborative Innovation Center for Food Production and Safety, College of Biological Science and Engineering, North Minzu University, Yinchuan, Ningxia 750021, People’s Republic of China; b School of Food and Biological Engineering, Hefei University of Technology, Hefei 230009, People’s Republic of China

**Keywords:** Ultrasound, Temperature, Starch–lipid complex, Chain length, Pea starch

## Abstract

Ultrasound is a promising green technology for modifying starch. The influence of ultrasound pretreatment (UPT) at diverse temperatures on the morphology and molecular structure of pea starch and its ability to form inclusion complexes with lipids were investigated. After UPT at each temperature, the starch granules retained an unchanged crystalline structure but exhibited notable changes in short-range molecular order and molecular structure. In comparison with the samples treated at 0 and 20 °C, pea starch subjected to UPT at 40 °C had a significantly (P ≤ 0.05) higher complexing index with lauric acid (LA) and the starch-LA inclusion complex exhibited a higher enthalpy change, relative crystallinity, and resistant starch content. These differences were attributed to the higher temperature causing changes in the disruption points of starch chains and an enlargement in the molecular weight of linear chains. These results may promote the utilization of ultrasound for effective starch modification.

## Highlights

• Ultrasound pretreatment (UPT) affected the morphology and structure of pea starch.
• The ultrasonic temperature altered the chain disruption points of pea starch.
• UPT at higher temperatures increased the number of linear chains and amylose content.
• UPT enhanced the complexation ability of pea starch with lauric acid.

## Introduction

Starch, a key component in many foods, has a considerable impact on the viscosity, moisture retention, consistency, texture, storage life, and digestive properties of foods after processing (Wang, Chao, et al., 2020). Owing to the specific gelatinization, pasting, and retrogradation attributes of starch, the quality characteristics of starchy foods are significantly affected by additives. Consequently, lipids are added to many starchy foods to optimize the quality and mouth feel (Wang, Wang, Yu, & Wang, 2016). During production, starch undergoes gelatinization and aging, and starch chains, especially linear chains, form clathrate complexes with exogenous or endogenous lipids. The generation of starch–lipid complexes can strongly influence the functional properties of food systems by reducing the swelling capacity and solubility as well as increasing the pasting temperature of starch samples in water, altering the rheological characteristics of starch paste, retarding retrogradation, and enhancing the resistance to enzymatic hydrolysis (Chao, Cai, et al, 2018). This complexation process is of considerable research interest because it can result in novel starch derivatives and has potential for the production of new delivery systems that prevent the volatilization and oxidation of polyunsaturated fatty acids, aromatic substances, and bioactive compounds (Kawai et al., 2012).

Extensive studies have revealed that starch–lipid complexation is influenced by numerous factors, including the starch characteristics (the content and chain length of amylose), lipid characteristics (concentration, saturation, and carbon chain length), and experimental conditions (Cai et al., 2021, Cai et al., 2021). In particular, the amylose content and chain length have notable effects on the formation of inclusion complexes. Guraya, Kadan, & Champagne (1997) reported that the complexation ability of lipids with waxy starch is much weaker than that with normal starch because the many short-branched chains in waxy starch prevent or restrict the formation of the helical structures required for lipid complexation. Furthermore, the amount of waxy wheat starch–lipid inclusion complexes formed is too few to be detected by differential scanning calorimetry (DSC) or X-ray diffraction (XRD) (Wang, et al, 2016). Liu, Kang, Cui, Gao, Wu, and Yu (2019) suggested that maize starch pretreated with pullulanase produces more amylose chains (degree of polymerization (DP) ≥ 25), which promotes the formation of maize starch–glycerol monolaurate inclusion complexes.

Pea starch is the main component (approximately 50%) of pea seeds grown worldwide and is extensively applied in industry (Hoover, Hughes, Chung, & Liu, 2010). Peach starch is distinguished by a high amylose content of 30–60% depending on the species, and thus has considerable potential for producing starch–lipid inclusion complexes. As pretreatment with maltogenic amylase and pullulanase increases the amylose content of pea starch, treated samples exhibit a higher capacity for forming starch–lipid inclusion complexes than native starch (Liu, Gao, et al, 2020). As an alternative starch modification method, ultrasound has the advantages of high efficiency, safety, eco-friendliness, and low cost. Ultrasound employs the energy generated by sound waves from 18 kHz to 1 GHz to stir up particles dispersed in solution. Ultrasound therapy can significantly (P ≤ 0.05) increase the content of amylose and alter the distribution of amylopectin chain lengths in pea starch (Han, Cao, et al., 2021). Additionally, Raza et al. (2021) found that treating mixtures of linoleic acid and arrowhead starch at various ultrasound frequencies can aid in the formation of starch–lipid inclusion complexes. However, there are no studies on the ability of ultrasound-pretreated pea starch to form starch–lipid inclusion complexes.

Ultrasound treatment can alter the characteristics of pea starch and thus influence complexation with lipids. This study focused on investigating the effects of ultrasound pretreatment (UPT) at various temperatures on the ability of pea starch to form the inclusion complexes between starch and lipid. The mechanistic insights obtained using a model system of pea starch and lauric acid (LA) provide an improved understanding of the complexation ability of ultrasound-treated starch.

## Materials and methods

### Materials

Pea starch (denoted as NS) was donated by Hebei Yizhilian Food Co., Ltd. The moisture, total starch, lipid, and protein contents of the starch sample were 10.30%, 89.00%, 0.52%, and 0.18%, respectively. Sodium acetate, 8-Aminopyrene-1,3,6-trisulfonic acid trisodium salt (APTS), petroleum ether, amyloglucosidase (A7420, 30–60 U/mg), pullulanase, porcine pancreatic α-amylase (10080, 50 U/mg), glucose oxidase/peroxidase reagent (G3660), and LA (C12:0) were obtained from Sigma Chemicals Co., Ltd., China. All other reagents and chemicals were of analytical grade.

### Preparation of starch samples and starch–lipid complexes

#### Ultrasound pretreatment (UPT)

Pea starch (10 g, dry weight basis) was dispersed in 100 mL of distilled water in a 150 mL Erlenmeyer flask. The starch suspension was treated using a JY98-IIIDN ultrasonic processor (LC-1200Y, Shanghai Bingyue Electronic Instrument Co., Ltd.) equipped with a probe with a tip diameter of 6 mm for 20 min (20 kHz, 300 W). The ultrasonic temperature was maintained at 0 °C by an ice bath and at 20 and 40 °C by circulating water. After centrifuging the mixture at 3000 × *g* for 10 min, the starch precipitate was dried at 40 °C to the constant weight and passed through a 100 mesh sieve. The starch samples subjected to UPT at 0, 20, and 40 °C were denoted as UPTS-0, UPTS-20, and UPTS-40, respectively.

#### Preparation of starch–lipid complex

Starch–lipid complexes were prepared using a rapid viscosity analyzer (RVA; TechMaster, Perten, Sweden). Each starch sample (2.20 g, dry weight basis) and 66.00 mg of LA were mixed with 25.734 g of distilled water in the special aluminum canister. The mixture was heated to 50 °C, held for 1 min, heated to 95 °C at a rate of 12 °C/min, held for 2.5 min, cooled to 50 °C at a rate of 12 °C/min, and held for 2 min. The prepared paste was immediately used to determine the complexing index (CI) or dried using an Alpha 2–4 LSC vacuum-reeze dryer (Martin Christ, Germany) for other analyses. The dried complexes of NS, UPTS-0, UPTS-20, and UPTS-40 with LA were denoted as NS-LA, UPTS-0-LA, UPTS-20-LA, and UPTS-40-LA, respectively.

### Morphology of NS and UPTS samples

#### Scanning electron microscopy (SEM)

The NS and UPTS samples were fixed on an aluminum stage and then coated with gold (K550X, Quorum Technologies Ltd., UK) and imaged at a magnification of 2000× using a JSM-6360LV scanning electron microscope (Hitachi S-3400, JEOL, Japan). The acceleration voltage was controlled at 5 kV.

#### Confocal laser scanning microscopy (CLSM)

NS or each UPTS sample (2 mg, dry weight basis) was mixed with sodium cyanoborohydride (1 M, 4 μL) and 10 mM APTS (4 μL). This mixture was then suspended in glycerol/water (1:1, v/v; 20 μL). A drop of the suspension was observed using a confocal laser scanning microscope (Nikon Co., Ltd., Tokyo, Japan) with a lens configuration of 100 × Plan apo/1.4 oil and 500–600 nm of the emission wavelength.

### Amylose content of NS and UPTS samples

The amylose contents of the NS and UPTS samples were analyzed using the method reported by Williams, Kuzina, and Hlynka (1970). The samples were degreased and analyzed using colorimetry.

### Molecular structure of NS and UPTS samples

#### Gel permeation chromatography (GPC)

The molecular weights of the NS and UPTS samples were determined using a GPC instrument (Model 410, Waters, USA) equipped with an HMW6E Styragel column (WAT044205, Waters, USA) and a differential refractive index detector (RI2414, Waters, USA). Each starch (10 mg dry basis) was dissolved in a solution (2 mL) of dimethyl sulfoxide (DMSO) containing LiBr (w/w, 0.5%) and then filtered through a filter (0.45 µm) before analysis. Dextran standards (Sigma-Aldrich Co., USA) with a wide range of molecular weights were used for calibration. The injection volume: 20 mL; the flow velocity of the mobile phase (DMSO/LiBr solution): 0.6 mL/min; the column temperature: 45 °C.

#### High-performance anion-exchange chromatography (HPAEC)

The distribution of amylopectin branch chain lengths in the NS and UPTS samples was analyzed by HPAEC with pulsed amperometric detection (ICS-5000+, Thermo Fisher Scientific, USA) referring to the report of Cai and Shi (2010) with minor modifications. An analytical column (CarboPac PA100) was used with 25 °C of the column temperature and 1 mL/min of the flow velocity. The mobile phase was a mixture of 150 mM NaOH (eluent A) and 150 mM NaOH containing 500 mM CH3COONa (eluent B). A gradient elution program with a total elution time of 60 min was as follows: 40% eluent B was maintained for 2 min, 50% eluent B was maintained for 8 min, 60% eluent B was maintained for 30 min, and 80% eluent B was maintained for 20 min, respectively.

### X-ray diffraction (XRD)

The XRD patterns of the starch samples and starch–lipid complexes were collected using an X-ray diffractometer (D/Max 2200, Japan) at a tube pressure of 40 kV and current of 100 mA. Measurements were performed in the 2θ range of 4°–60° with a scanning speed of 6°/min and a step size of 0.02°. For the NS and UPTS samples, the relative crystallinity (RC) was calculated using Equation (1):

(1) $$RC\left(\%\right)=\frac{A_{c}}{A_{c+A_{a}}}\times 100$$

where *A_c_* indicates the peak areas of the crystalline regions and *A_a_* of the amorphous regions in the XRD patterns. The RC value of each starch–lipid complex was calculated using the TOPAS 5.0 software (Bruker, Germany).

### Laser confocal micro-Raman (LCM-Raman) spectroscopy

Short-range molecular order in the NS and UPTS samples and the starch–lipid complexes was investigated using a Raman microscope system (Renishaw Invia, UK) equipped with a green diode laser source (785 nm). Spectra were recorded in the range of 3200–100 cm^−1^ at a resolution of approximately 7 cm^−1^. To reflect the short-range molecular order of each sample, the full width at half maximum (FWHM) of the band at 480 cm^−1^ was determined using Origin 8.5 software.

### Differential scanning calorimetry (DSC)

The thermal characteristics of the NS and UPTS samples and the starch–lipid complexes were evaluated using a 214F_3_ differential scanning calorimeter (Netzsch, Germany). Each sample (3.00 mg, dry basis weight) was placed in an aluminum pan with 12.00 μL of distilled water. The pan was sealed, allowed to equilibrate for 12 h at 25 °C, and then heated from 20 to 120 °C (10 °C/min). An identical empty aluminum pan was utilized as a reference.

### Complexing index (CI) analysis

The paste of each starch–LA complex prepared using the RVA procedure (5.0 g) was added to 25 mL of ultrapure water, preheated to the corresponding temperature at the end of the RVA procedure, and vortexed for 1 min. The prepared suspension (50 μL) was added with the ultrapure water (15 mL) and iodine solution (2 mL, aqueous solution containing 2.0% KI and 1.3% I_2_) and then vortexed. The absorbance of this mixture was recorded at 620 nm. Pastes containing only starch prepared using the RVA procedure were used as references. The CI value was calculated according to the following equation:

(2) $$CI\;\left(\%\right)=\frac{A_{P}-A_{C}}{A_{P}}\times 100$$

where *A_P_* and *A_C_* indicate the absorbance of the paste of the pure starch and the starch–lipid complex.

### In vitro digestibility

The in vitro digestibility of the NS and UPTS samples and the starch–lipid complexes was analyzed using the method provided by Englyst, Kingman, and Cummings (1992) with minor modifications. Porcine pancreatic α-amylase (1 g) was dispersed in 16.3 mL of deionized water, magnetically stirred for 30 min, and centrifuged for 10 min (1500 × *g*). The supernatant fluid was collected as solution A (α-amylase solution). Amyloglucosidase (100 mg) was suspended in 1 mL of deionized water, magnetically stirred (30 min), centrifuged at 1500 × *g* (10 min). The supernatant was collected as solution B (amyloglucosidase solution). Then, 0.1 mL of solution A was mixed with 8 mL of solution B to obtain the enzyme solution. Each sample was dispersed in 0.1 M sodium acetate buffer (pH 5.2, 25 mL), mixed with the fresh enzyme solution (5 mL), and incubated at 37 °C with continuous stirring (200 rpm). Aliquots (0.1 mL) removed from the sample solution at intervals of 20 and 120 min were added to 1 mL of 90% ethanol and centrifuged for 5 min (3000 × *g*). The obtained supernatants were used to determine the glucose content using a glucose oxidase/peroxidase reagent. The hydrolyzed starch content in the sample was obtained by multiplying the amount of glucose by a conversion factor of 0.9.

### Statistical analysis

Each experiment was repeated three times, and the reported results were processed using the SPSS statistical software package (10.1, SPSS Inc., USA). Significant differences were calculated using analysis of variance (ANOVA) with 0.05 of significance level. Graphs were prepared using Origin 8.0 software.

## Results and discussion

### Morphology of NS and UPTS samples

**Fig. 1:**
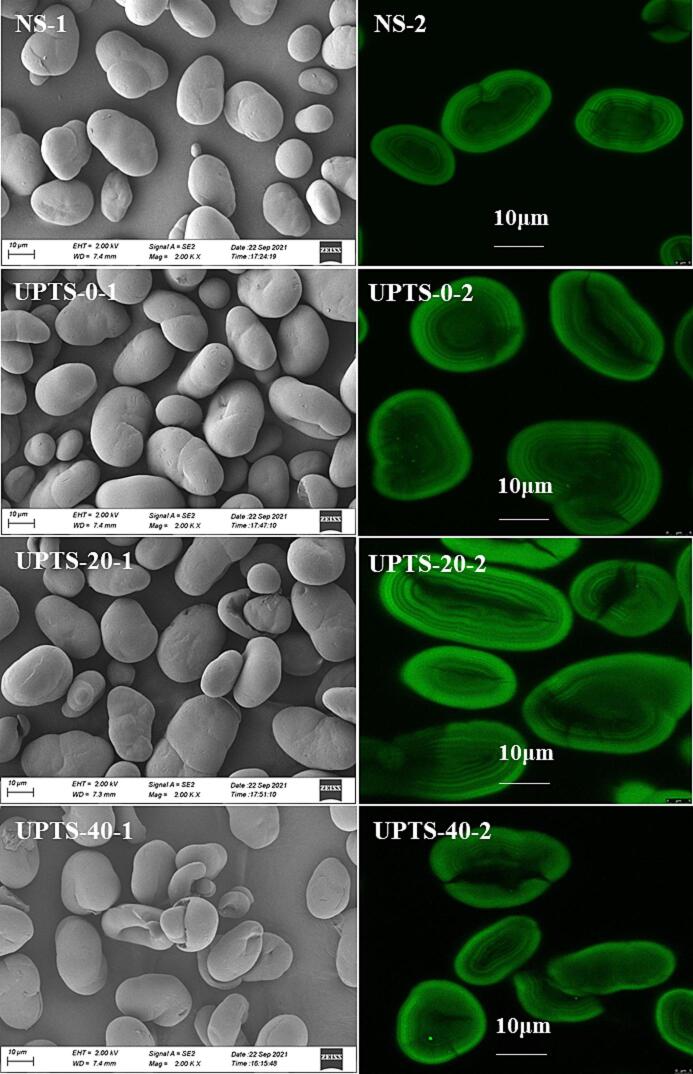
Scanning electron micrographs (SEM) (2000 × ) (1) and confocal laser scanning micrographs (CLSM) (2) of native and ultrasound treated pea starches.

SEM images of NS and UPTS granules at 2000 × magnification are shown in Fig. 1. The NS granules have an oval-like morphology and a smooth surface without obvious cracks or grooves (Fig. 1, NS-1). No noticeable morphological changes are observed for the starch samples subjected to UPT at 0 °C (Fig. 1, UPTS-0–1) and 20 °C (Fig. 1, UPTS-20–1), but the starch granules treated at 40 °C have obvious holes or plicated surfaces. Han et al. (2021) reported that ultrasound therapy (300 W, 20 kHz, 20 min, and 0 °C) does not change the surface morphology of pea starch granules. However, profound cavities and fractures have been observed on the surfaces of plantain and taro starch granules after ultrasound treatment (80 W, 25 kHz, 20 or 50 min) (Carmona‐García, Bello‐Pérez, et al., 2016). These results show that the impact of UPT on the surface structure of starch granules is dependent on the treatment conditions and the sample origin.

CLSM images of APTS-stained starch granules are shown in Fig. 1. The reducing ends of starch molecules can react with the APTS colorant and fluoresce. The reducing ends of NS are mainly located in the circumgranular region, as demonstrated by the fluorescence intensity being higher in the periphery of the starch particles than in the umbilicus region (Fig. 1, NS-2), consistent with the findings of Han, Cao, et al. (2021). The starch umbilical-region is encircled by growth rings, which are the inner ring layers generated by the alternating arrangement of amorphous layers and crystalline layers (Su et al., 2020). The CLSM results reveal a notable change in the reducing-end distribution and growth-ring structure of pea starch particles after UPT (Fig. 1, UPTS-0-2, UPTS-20-2, and UPTS-40-2). Thus, UPT at different temperatures can alter the chain distribution and granule structure of pea starch.

### Amylose content of NS and UPTS samples

The amylose contents of the NS and UPTS samples are listed in Table 1. UPT at different temperatures significantly (P ≤ 0.05) increases the amylose content in pea starch, which is consistent with previous reports of ultrasound treatment increasing the amylose content of sago, maize, pea, mung bean, and potato starch samples (Chan et al., 2010, Han et al., 2021). These changes are because of the partial chain-depolymerization of starch by UPT, which increases the linear chain and amylose content in the starch particles. The amylose content of UPTS-40 is significantly (P ≤ 0.05) higher than that of UPTS-0 and UPTS-20. Ultrasound treatment at 60 °C has been shown to induce a significant (P ≤ 0.05) decrease in the swelling capacity of potato and millet starch compared with ultrasound treatment at 25 °C, likely because the high-temperature treatment causes structural changes in starch granules (Hu, Li, & Zheng, 2019).

**Table 1:** Amylose content, molecular weight, chain length distribution of amylopectin, and complexing index with LA of native and ultrasound-pretreated pea starch.a, b.

Sample	Amylose content (%)	Complexing index (%)	Molecular weight(g/mol)		Chain length distributions (%)			
			Peak 1 (×10^5^)	Peak 2 (×10^3^)	DP6-12	DP13-24	DP25-36	DP > 37
NS	35.72 ± 0.67c	22.53 ± 1.02a	22.13 ± 0.08a	44.65 ± 0.09c	17.80 ± 0.27a	46.43 ± 0.45a	23.96 ± 0.56b	11.82 ± 0.46d
UPTS-0	36.96 ± 0.36b	26.17 ± 0.98b	19.89 ± 0.09b	45.87 ± 0.10b	20.51 ± 0.75b	49.18 ± 0.31b	22.35 ± 0.28a	9.97 ± 0.16c
UPTS-20	37.39 ± 0.42b	27.32 ± 1.15b	19.72 ± 0.10b	45.69 ± 0.09b	20.08 ± 0.77b	50.40 ± 0.63b	24.13 ± 0.87b	5.40 ± 0.11b
UPTS-40	38.99 ± 0.91a	34.66 ± 1.45c	18.45 ± 0.12c	46.72 ± 0.08a	19.00 ± 0.21b	50.77 ± 0.40b	26.16 ± 0.33c	4.08 ± 0.13a

^a^q, the scattering vector; d, the thickness of semi-crystalline lamellae; da, the thickness of amorphous lamellae; dc, the thickness of crystalline lamellae; α, the power-law exponent.

^b^Different letters within the same column for the same type samples show a significantly different (p ≤ 0.05). All results are means of triplicate determinations ± standard deviation.

### Molecular structure of NS and UPTS samples

#### Molecular weight

The molecular weights of the NS and UPTS samples are summarized in Table 1. Two components were observed in the high-performance gel chromatograms: one is a smaller molecular weight corresponding to amylose and other low-molecular-weight molecules, and the other is a larger molecular weight corresponding to amylopectin molecules. Compared with NS, the UPTS samples exhibit a significantly (P ≤ 0.05) lower molecular weight of amylopectin and a higher molecular weight of amylose. This result confirms that UPT contributes to the degradation of starch molecules with high molecular weights and the production of molecules with low molecular weights. This trend is consistent with the finding that among polymer degradation methods, ultrasound has a greater tendency to degrade larger polymers (Vodeničarová, Dřímalová, Hromádková, Malovíková, & Ebringerová, 2006), as demonstrated by several previous reports (Han et al., 2021, Wang et al., 2020).

Moreover, there are no significant (P > 0.05) differences in the molecular weights of amylose and amylopectin between UPTS-0 and UPTS-20. However, UPTS-40 has a significantly (P ≤ 0.05) lower molecular weight of amylopectin than the other UPTS samples, whereas the opposite trend is observed for the molecular weight of amylose. These results indicate that amylopectin chain degradation induced by UPT is enhanced at 40 °C. It has been found that temperatures higher than 70 °C remarkably reduce the cavitation power of ultrasound (Raso, Manas, Pagan, & Sala, 1999) and hinder the influence of ultrasonic cavitation on starch granules (Wang et al., 2017). Nevertheless, a temperature of 40 °C may enhance the UPT effects on starch structure by decreasing the stability of starch granules.

#### Amylopectin branch chain length distribution

The distributions of amylopectin chain lengths in the NS and UPTS samples are summarized in Table 1. The report of Hanashiro, Abe, and Hizukuri (1996) indicated that amylopectin side chains can be classified into four types according to their DP: A chains with DP of 6–12, B_1_ chains with DP of 13–24, B_2_ chains with DP of 25–36, and B_3_ chains with DP of 37. The amylopectin chain length distribution of NS showed a maximum for B_1_ chains. UPT at 0 °C induced a significant (P ≤ 0.05) shift of the branch chain length distribution toward shorter chains, resulting in UPTS-0 having a significantly (P ≤ 0.05) higher proportion of A and B_1_ chains than NS, consistent with previous findings (Han, Cao, et al., 2021). It has been found that ultrasound treatment has a better capacity to destroy branched amylopectin chains close to the branching points than amylose chains (Wang et al., 2020). The B chains of amylopectin, which are internal chains, are the main component of amorphous flakes (Bertoft, Piyachomkwan, Chatakanonda, & Sriroth, 2008). Kim et al. (2012) found that the internal chains in the amorphous region are the dominant disruption sites in starch granules. Therefore, UPT induces the depolymerization of amylopectin chains and enhances the proportion of A and B_1_ chains.

However, UPT at 20 and 40 °C had a different impact on the branch chain length distribution compared with UPT at 0 °C. The contents of B_2_ chains in UPTS-20 and UPTS-40 are significantly (P ≤ 0.05) higher than that in UPTS-0, and UPTS-40 contains significantly (P ≤ 0.05) more B_2_ chains than UPTS-20. These findings can be explained by a change in the disruption points of starch chains at high temperatures, with disruption preferentially occurring at the B_3_ chains of amylopectin under higher ultrasonic temperatures (Wei, Pu, et al., 2022).

### Long-range order in starch samples and starch–lipid complexes

**Fig. 2:**
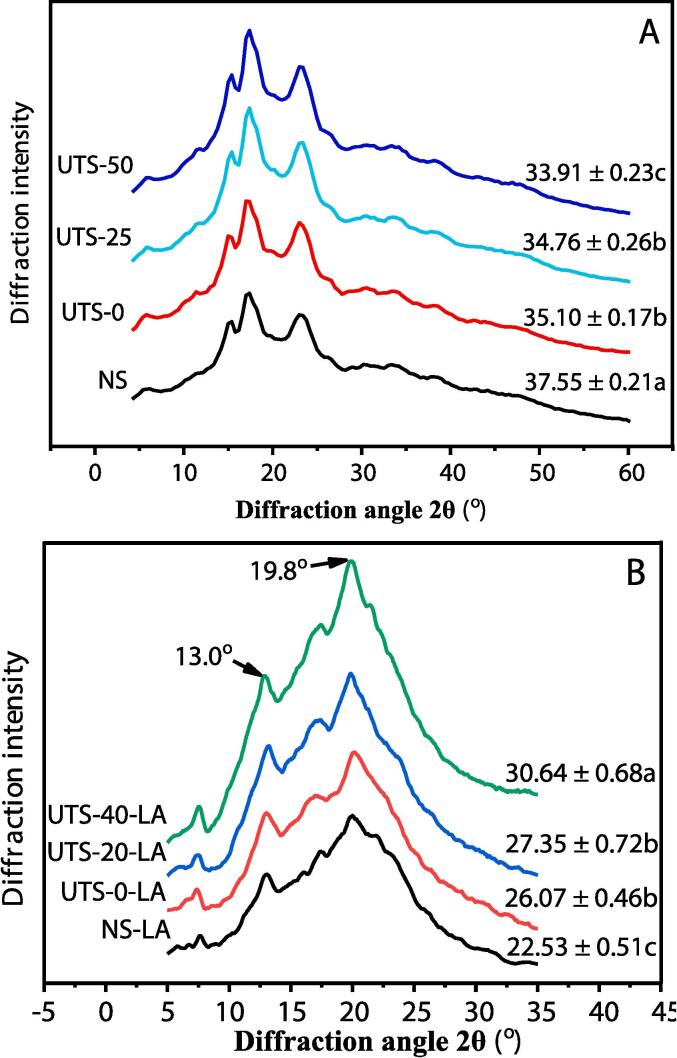

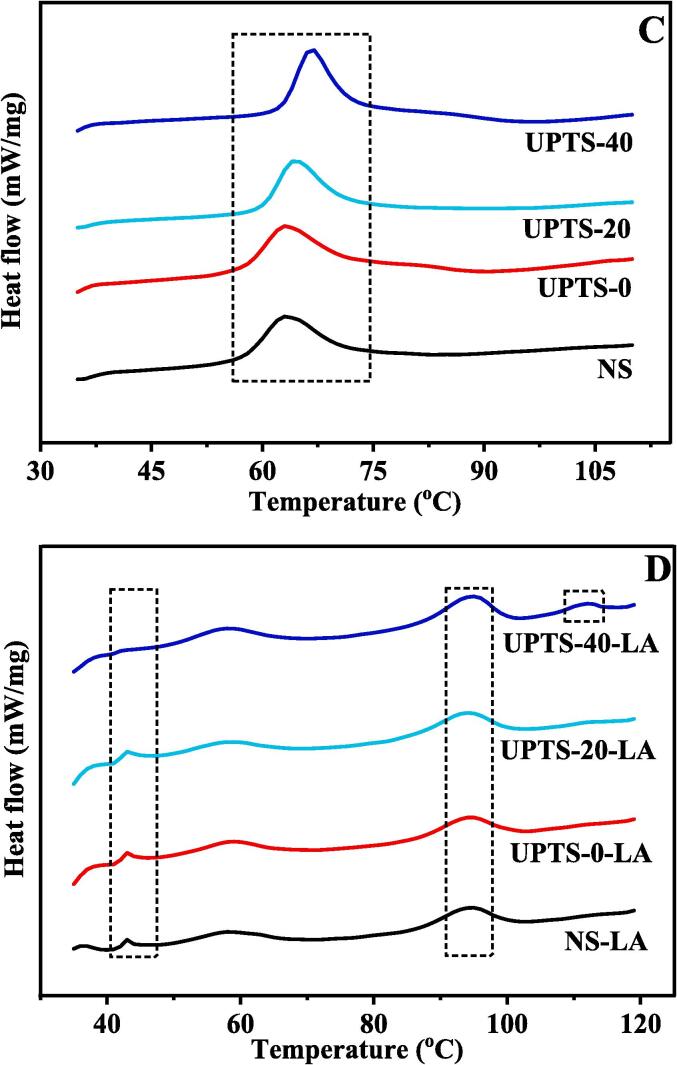
XRD patterns (A, B) and DSC thermograms (C, D) of pea starches and starch-lipid inclusion complexes. The relative crystallinity values reported in the XRD patterns indicate the mean ± standard deviation of triplicates; different letters within the same column for the same type samples show a significantly difference (p < 0.05).

NS exhibits a typical C-type diffraction pattern with single crystalline peaks at 17–18° and 22–24° (Fig. 2A). UPT did not alter the C-type structure of pea starch. This finding is in agreement with previous reports (Han et al., 2021, Kunyanee and Luangsakul, 2020). The diffraction patterns of the starch–lipid complexes show a crystalline structure with prominent peaks at 13.0° and 19.8° (Fig. 2B), manifesting the formation of inclusion complexes between LA and pea starch (Wang et al., 2020, Zhang et al., 2012, Zhang et al., 2022).

The RC values of the starch samples and starch–lipid complexes are shown in Fig. 2. The RC values of the pea starch granules (Fig. 2A) decrease significantly (P ≤ 0.05) after UPT. In addition, as the ultrasonic temperature increases, the RC values decrease significantly (P ≤ 0.05). These results indicate that UPT induces the destruction of the crystal structure of starch particles, which is in agreement with the CLSM results. Potato and millet starch have been reported to show decreased RC values after UPT (40 kHz or 40 + 80 kHz) at 25 and 60 °C (Hu et al., 2019). Similarly, the RC values of pea starch have decreased after ultrasound treatment (20 kHz, 0 °C) (Han, Cao, et al., 2021). In contrast, wheat starches exhibit higher RC values after ultrasound treatment (30 kHz) (Karwasra, Kaur, & Gill, 2020). Which demonstrate that the influence of UPT on the RC values of starch granules depends on the starch origin and treatment conditions. Differences in the packing of crystalline and amorphous regions endow starch particles with diverse susceptibilities to ultrasound treatment (Hu et al., 2019).

The RC values of the UPTS-LA complexes (Fig. 2B) are greater than that of the NS-LA complex, indicating that ultrasound-pretreated pea starch favors the formation of starch–LA inclusion complexes, which is attributable to the higher amylose content. The enzymatic hydrolysis of amylopectin has been reported to promote the generation of starch–lipid complexes by increasing the content of linear amylose in starch samples (Cai, Chao, et al., 2021). In addition, increasing the ultrasonic temperature significantly (P ≤ 0.05) increases the RC values of the UPTS-LA complexes. The higher ultrasonic temperature may enhance the mobility of the amylose linear chains with medium molecular weights and increase the average linear chain length in ultrasound-pretreated starch. Linear starch chains with a DP of more than 20 can more easily form a V-type crystalline structure with LA than other starch chains (Zhang et al., 2023).

### Short-range molecular order in starches and starch–lipid complexes

**Fig. 3:**
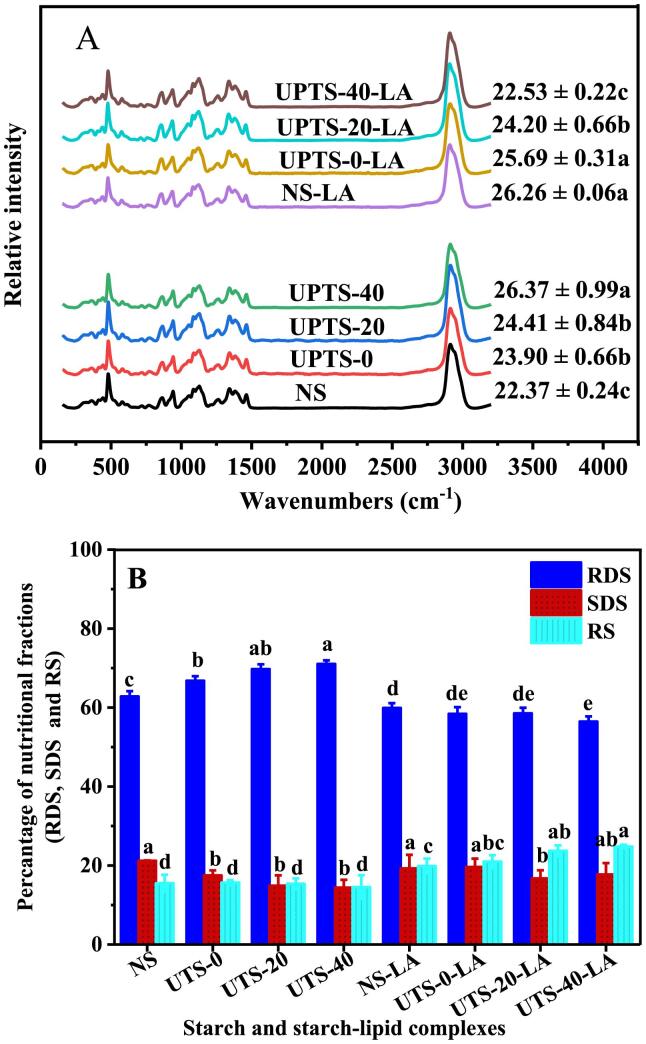
LCM-Raman spectra and the nutritional fractions (RDS, SDS, and RS) of pea starch samples and starch-LA inclusion complexes. The FWHM values reported in the LCM-Raman spectra indicate the mean ± standard deviation of triplicates; different letters within the same column for the same type samples show a significantly difference (p < 0.05). Each plotted point of RDS, SDS, and RS in an average of three trials ± SD; values followed by the same letters in the same bar of the specific starch fraction do not differ significantly at p < 0.05 level.

Fig. 3A shows the LCM-Raman spectra of the NS and UPTS samples and the starch–lipid complexes as well as the corresponding FWHM values of the band at 480 cm^−1^. The FWHM value is negatively correlated to the short-range order of the starch molecular structure (Chao et al., 2018, Liu et al., 2021). The FWHM values of the UPTS samples are remarkably (P ≤ 0.05) higher than that of NS, and UPTS-40 has a significantly (P ≤ 0.05) higher FWHM value than UPTS-20 and UPTS-0. These results indicate that UPT induces the destruction of short-range molecular order in pea starch, and this effect is amplified at higher temperatures. Ultrasound treatment has also been found to reduce the short-range order of kiwi and sweet potato starches (Wang et al., 2020, Wang et al., 2022). Ultrasound treatment may change the crystalline and amorphous regions of starch granules, contributing to an expansion or irregular arrangement of spiral structures.

The UPTS-20-LA and UPTS-40-LA complexes have significantly (P ≤ 0.05) lower FWHM values than NS-LA and UPTS-0-LA. This result demonstrates that UPT at 20 and 40 °C enhances the complexation ability of pea starch with lipids and promotes the formation of molecular structures with short-range order, consistent with the XRD results.

### Thermal characteristics of starch samples and starch–lipid complexes

DSC thermograms of the NS and UPTS samples and the starch–lipid complexes are shown in Fig. 2C, D and the corresponding thermal properties are summarized in Table 2. Statistical analysis indicates that UPT at 0 °C does not significantly influence the thermal parameters of pea starch, which is in accordance with previous reports for A. squarrosum starch (Han, Wei, et al., 2021). However, UPT at 20 and 40 °C remarkably (P ≤ 0.05) increased the onset temperature (To) and peak temperature (Tp) of UPTS-20 and UPTS-40. This behavior might be due to structural recombination, which hinders the mobility of starch chains in the amorphous lamella. In addition, UPT significantly reduces the enthalpy change (ΔH) of pea starch. The ΔH value has been proposed to indicate the amount and quality of crystalline structures in starch particles. Thus, the decreased ΔH values of the UPTS samples demonstrate that UPT decreases the stability of starch crystals by altering the lamellar structure and even the chain structure.

**Table 2:** Thermal properties of pea starch samples and starch–lipid complexes.^a,b^

Sample	Thermal properties			
	To (℃)	Tp (℃)	Tc (℃)	ΔH (J/g)
NS	57.42 ± 0.22c	63.31 ± 0.11c	70.8 ± 0.30a	14.22 ± 0.19a
UPTS-0	57.34 ± 0.25c	63.65 ± 0.72c	70.3 ± 1.72a	12.94 ± 0.47b
UPTS-20	60.06 ± 0.50b	64.34 ± 0.26b	71.1 ± 0.32a	12.74 ± 0.55b
UPTS-40	62.88 ± 0.56a	66.73 ± 0.33a	71.2 ± 0.45a	13.82 ± 0.36b

NS-LA	86.3 ± 1.0a	94.12 ± 0.41a	99.2 ± 0.66a	3.23 ± 0.13d
UPTS-0-LA	85.3 ± 0.3a	94.07 ± 0.14a	99.3 ± 0.80a	4.62 ± 0.3c
UPTS-20-LA	85.7 ± 0.7a	93.40 ± 0.42a	99.8 ± 0.43a	6.15 ± 0.31b
UPTS-40-LA	84.8 ± 0.8a	94.19 ± 0.61a	99.5 ± 0.15a	7.77 ± 0.69a

^a^To, Tp, Tc, and ΔH indicate the onset temperature, peak temperature, conclusion temperature and enthalpy of gelatinization.

^b^Different letters within the same column for the same type samples show a significantly difference (p ≤ 0.05). All results are means of triplicate determinations ± standard deviation.

The DSC thermograms of NS-LA, UPTS-0-LA, and UPTS-20-LA (Fig. 2D) exhibit a weak peak at approximately 40 °C, which is because of the melting of excess LA in the samples (Chao, Cai, et al, 2018). However, this peak is not observed for UPTS-40-LA, indicating a higher degree of complexation between UPTS-40 and LA than between NS, UPTS-0, or UPTS-20 and LA. In addition, NS-LA, UPTS-0-LA, and UPTS-20-LA show a single endothermic peak at approximately 95 °C, indicating that the samples only contain type I complexes. In contrast, UPTS-40-LA exhibits two peaks at approximately 95 and 115 °C, consistent with the presence of both type I and type II complexes (Zhang et al., 2012). Type I complexes are less ordered, whereas type II complexes are lamellar and more stable (Karkalas, Ma, Morrison, & Pethrick, 1995). The linearity and length of starch chains are the most influential structural factors on the generation and properties of starch–lipid inclusion complexes, and longer linear chains tend to form stable complexes (Zhang et al., 2023). The difference in the endothermic peaks among the NA-LA and UPTS-LA complexes might be due to the retention or production of longer linear chains in pea starch at higher ultrasonic temperatures. Furthermore, the ΔH values of the UPTS-LA samples are remarkably (P ≤ 0.05) higher than that of NS-LA, and they enhance significantly (P ≤ 0.05) with increasing ultrasonic temperature, indicating that UPT at higher temperatures can promote the formation of ordered structures. This behavior is consistent with the LCM-Raman and XRD results.

### CI analysis

The CI is a measure of the ability of amylose to bind to iodine, and a high CI value indicates a high value of binding index between amylose and lipids. The UPTS-LA complexes have remarkably (P ≤ 0.05) higher CI values than NS-LA (Table 1), and that the CI value of UPTS-40-LA is significantly (P ≤ 0.05) higher than those of UPTS-20-LA and UPTS-0-LA. A previous report indicated that the content of amylose is a key factor that affects the CI value of legume starches (Oyeyinka, Singh, Ma, & Amonsou, 2016). A high content of linear chain segments (amylose) in starch results in a high CI with lipids.

### In vitro digestibility of starch samples and starch–lipid complexes

Based on their digestibility in humans, starches can generally be classified as three categories: rapidly digested starch (RDS), slowly digested starch (SDS) and resistant starch (RS), respectively. The amounts of RDS, SDS, and RS in the NS and UPTS samples and the starch–lipid complexes are exhibited in Fig. 3B. UPT remarkably (P ≤ 0.05) reduces the SDS content and increases the RDS content of pea starch, which is in line with the previous founding of Han et al. (2021). Sullivan, Pangloli, and Dia (2017) reported that ultrasound treatment can induce the depolymerization of starch chains and attenuate the interactions between proteins and starch, thus accelerating starch hydrolysis and increasing the RDS content of ultrasound-treated samples (Sullivan, Pangloli, & Dia, 2017). The RDS content of UPTS-40 is remarkably (P ≤ 0.05) higher than those of the other UPTS samples, indicating that heat can enhance the effect of ultrasound therapy on starch granules.

The starch–lipid complexes contain significantly (P ≤ 0.05) higher amounts of RS and lower amounts of RDS than the corresponding starch samples. Thus, the formation of starch–lipid complexes increases starch resistance to enzymatic hydrolysis, as previously reported (Wang, Chao, et al, 2020). Conformational hindrance within starch–lipid complexes restricts the access of enzymes associated with digestion to starch granules (Putseys, Lamberts, & Delcour, 2010).

In addition, the RS content of UPTS-40-LA is significantly (P ≤ 0.05) higher than those of NS-LA and UPTS-0-LA, indicating that UPTS-40-LA has a higher CI than the other samples. This result is consistent with the observed variation in the calculated CI values.

## Conclusion

UPT at various temperatures (0, 20, and 40 °C) was found to induce differences in the granule, crystalline, and molecular structure of pea starch, and its ability to form inclusion complexes with lipids. The starch granules retained the native crystalline structure of C-type after UPT at all temperatures but the relative crystallinity, short-range molecular order, and amylopectin molecular weight decreased with increasing treatment temperature. UPT enhanced the complexation ability of pea starch with LA, and the sample treated at 40 °C possessed a remarkably higher (P ≤ 0.05) CI than those treated at 0 and 20 °C because the higher temperature changed the breaking points of the starch chains and modulated the distribution of linear chain lengths. These results can provide theoretical guidance for the use of ultrasound technology to produce modified starch materials.

## CRediT authorship contribution statement

**Liuyang Xiao:** Investigation, Formal analysis, Data curation, Visualization, Writing – original draft, Writing – review & editing. **Yingtao Yu:** Investigation, Formal analysis, Data curation, Writing – original draft. **Xiaofan Yang:** Investigation, Formal analysis, Data curation, Writing – original draft. **Zhaojun Wei:** Visualization, Writing – review & editing. **Lihong Han:** Conceptualization, Methodology, Validation, Writing – original draft, Writing – review & editing.

## Declaration of Competing Interest

The authors declare that they have no known competing financial interests or personal relationships that could have appeared to influence the work reported in this paper.

## Data Availability

Data will be made available on request.
